# Extensive Loss and Gain of Conserved Noncoding Elements During Early Teleost Evolution

**DOI:** 10.1093/gbe/evae061

**Published:** 2024-04-22

**Authors:** Elisavet Iliopoulou, Vasileios Papadogiannis, Costas S Tsigenopoulos, Tereza Manousaki

**Affiliations:** Hellenic Centre for Marine Research (HCMR), Institute of Marine Biology, Biotechnology & Aquaculture (IMBBC), Heraklion, Greece; Present Address: Université Paris Cité, CNRS, Institut Jacques Monod, F-75013 Paris, France; Hellenic Centre for Marine Research (HCMR), Institute of Marine Biology, Biotechnology & Aquaculture (IMBBC), Heraklion, Greece; Present Address: Center for Genomic Regulation, Barcelona Institute of Science and Technology, Barcelona, Spain; Hellenic Centre for Marine Research (HCMR), Institute of Marine Biology, Biotechnology & Aquaculture (IMBBC), Heraklion, Greece; Hellenic Centre for Marine Research (HCMR), Institute of Marine Biology, Biotechnology & Aquaculture (IMBBC), Heraklion, Greece

**Keywords:** CNE, conserved noncoding elements, whole-genome duplication, enhancer evolution, teleost comparative genomics

## Abstract

Conserved noncoding elements in vertebrates are enriched around transcription factor loci associated with development. However, loss and rapid divergence of conserved noncoding elements has been reported in teleost fish, albeit taking only few genomes into consideration. Taking advantage of the recent increase in high-quality teleost genomes, we focus on studying the evolution of teleost conserved noncoding elements, carrying out targeted genomic alignments and comparisons within the teleost phylogeny to detect conserved noncoding elements and reconstruct the ancestral teleost conserved noncoding elements repertoire. This teleost-centric approach confirms previous observations of extensive vertebrate conserved noncoding elements loss early in teleost evolution, but also reveals massive conserved noncoding elements gain in the teleost stem-group over 300 million years ago. Using synteny-based association to link conserved noncoding elements to their putatively regulated target genes, we show the most teleost gained conserved noncoding elements are found in the vicinity of orthologous loci involved in transcriptional regulation and embryonic development that are also associated with conserved noncoding elements in other vertebrates. Moreover, teleost and vertebrate conserved noncoding elements share a highly similar motif and transcription factor binding site vocabulary. We suggest that early teleost conserved noncoding element gains reflect a restructuring of the ancestral conserved noncoding element repertoire through both extreme divergence and de novo emergence. Finally, we support newly identified pan-teleost conserved noncoding elements have potential for accurate resolution of teleost phylogenetic placements in par with coding sequences, unlike ancestral only elements shared with spotted gar. This work provides new insight into conserved noncoding element evolution with great value for follow-up work on phylogenomics, comparative genomics, and the study of gene regulation evolution in teleosts.

**Significance**

Thousands of conserved noncoding elements (CNEs) that have been conserved across 400 million years of vertebrate evolution were lost in teleost fish, through a process largely enigmatic to date. Here we use recent high-quality genomes to explore how CNEs evolved during fish evolution, confirming rapid loss and divergence in stem-teleosts, but also showing extensive fast gain of thousands of CNEs. We show these elements emerged around similar development associated loci and they share highly comparable transcription factor binding site composition as other vertebrate CNEs. This work supports that early teleost CNE gains may reflect a restructuring of the ancestral CNE repertoire through both extreme divergence and de novo emergence.

## Introduction

During embryonic development, the interaction of a network of transcription factors (TFs) with a variety of *cis*-regulatory elements found mainly in the noncoding part of the genome drives the spatial and temporal regulation of gene expression with great precision (Davidson 2010). In vertebrates, regulatory elements can be close to target genes (<50 kbp) or even embedded in intronic areas, but many *cis*-regulatory modules are located from hundreds of kbp to more than 1 Mb away (Jeong et al. 2006). Among the noncoding genome, a large number of vertebrate conserved noncoding elements (CNEs) have maintained greater than 70% sequence identity for over 400 million years, exhibiting even greater average conservation than protein-coding genes (Polychronopoulos et al. 2017). Many of these CNEs act as enhancers for neighboring genes associated with evolutionarily conserved functions, including important developmental processes (Woolfe et al. 2005). Most vertebrate CNEs are found in a single copy in the genome, but some CNE duplications have also been identified. These are found in paralogous loci produced by the two rounds (2R event) of whole-genome duplication (WGD) that happened early in vertebrate evolution, which have been suggested to have contributed to the diversification of the subphylum (Soukup 1974).

While jawed vertebrates share thousands of CNEs, a few hundred of these elements, including duplicate CNEs, are even conserved with lamprey, a member of the basally splitting jawless vertebrates (Papadogiannis et al. 2022). Therefore, the evolution of a set of ancestral vertebrate CNEs predates the split of jawless and jawed vertebrates, possibly dating to the first WGD shared by both lineages, with additional CNEs established after the second WGD of jawed vertebrates. Within vertebrates, teleost fish form the most species-rich monophyletic group (Nelson et al. 2016). It is hypothesized that one of the main drivers of their widespread diversification is the teleost-specific (3R event) WGD (Glasauer and Neuhauss 2014). Because of 3R, teleost genomes have increased genetic diversity, carrying additional paralogous genes. Unexpectedly, in contrast to gene coding regions, earlier reports have suggested extensive loss and rapid divergence of ancestral vertebrate CNEs following the 3R WGD event (Lee et al. 2011; Glasauer and Neuhauss 2014), though this has been based on few model species and comparisons to other vertebrates. To date, the drivers of this massive loss of CNEs after 3R have been unexplored, while the conservation of noncoding elements within the teleost clade has remained uncharacterized.

Previous studies on teleost CNE evolution had mainly focused on the fate of ancestral vertebrate CNEs in teleost fish (Lee et al. 2011). Therefore, a comprehensive catalogue of noncoding elements conserved within the teleost group is lacking and the way the CNE landscape responded to the 3R WGD remains unresolved. Here, we focus on identifying and studying teleost CNEs through a teleost-centric search, with the main focus of investigating the evolution of CNEs following the 3R WGD. For this purpose, we carry out targeted genome alignments of key focal species and use these data to detect CNEs across the teleost phylogeny. Drawing information of CNE presence and absence in different teleost groups, we reconstruct the ancestral teleost CNE repertoire and test the potential of these ancestral teleost CNEs as markers for resolving phylogenetic teleost placements. We then assess sequence and synteny conservation of teleost CNEs and use an orthology-guided synteny-based approach to associate CNE gains and losses with candidate conserved gene targets with putative regulatory links to studied CNEs. Finally, we use motif discovery and enrichment analysis to compare the TF-binding site composition of gained and ancestral CNEs. This work represents the first CNE analysis on a large number of teleost genomes and as such it provides unique insight for understanding the evolution of the conserved noncoding genome of teleosts.

## Results

### Teleost CNE Identification and Ancestral set Reconstruction

For the de novo identification of teleost CNEs, we first carried out whole-genome pairwise alignments in two pairs of focal reference species with high-quality annotations in selected positions of the phylogeny: (1) Zebrafish vs Mexican tetra and (2) Fugu vs gilthead seabream. After filtering out coding and repetitive regions, this investigation resulted in a set of 298,743 conserved sequence chains between Zebrafish and Mexican tetra and 372,053 chains between Fugu and gilthead seabream, yielding a total of 63,023 Zebrafish–Tetra CNEs (zCNE) and 39,532 Fugu–Seabream CNEs (fCNE) of at least 100 bp with >=70% sequence identity.

**Fig. 1.**
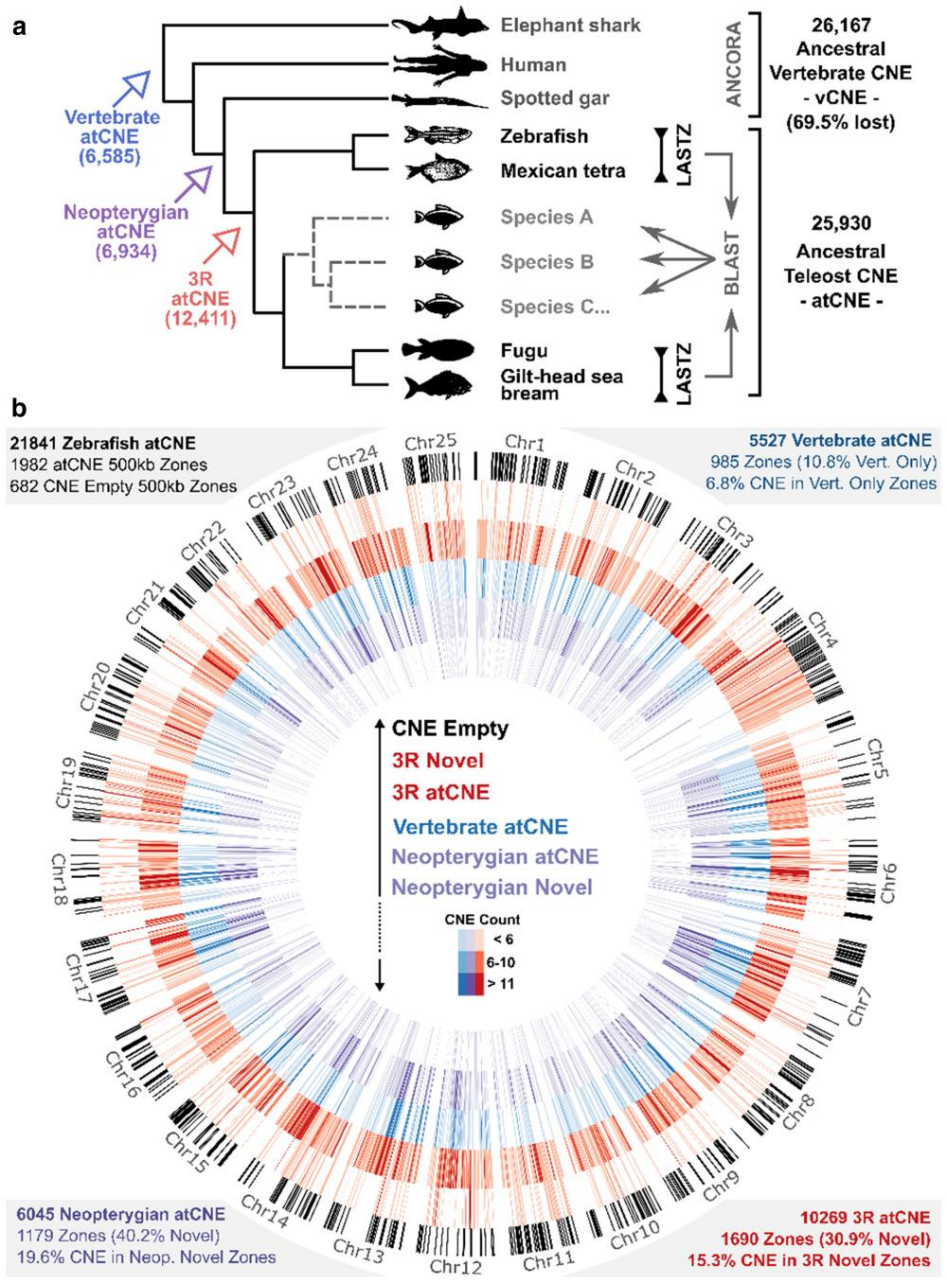
Teleost ancestral CNE identification and distribution. a) CNE sets from pairwise whole-genome alignments in two focal reference genome pairs (LASTZ) were searched in 20 other teleost genomes via BLAST, identifying 25,930 atCNEs that were present in the teleost common ancestor. Comparison to nonteleost vertebrate genomes and ancestral vertebrate CNEs from the ANCORA database revealed widespread loss of vertebrate atCNEs and gain of novel atCNEs in Neopterygians and Teleosts (3R). b) Distribution and count of 21,841 atCNEs over 1,982 500 kb-long zones in the zebrafish genome. From largest (outermost) to smallest (innermost) circle radius: (1) Genomic regions with no atCNE presence (black). (2) Novel zones with 3R atCNE presence (red) and absence of older (Neopterygian or vertebrate) atCNE. (3) Zones with 3R atCNE presence (red). (4) Zones with vertebrate atCNE presence (blue). (5) Zones with Neopterygian atCNE presence (purple). (6) Novel zones with Neopterygian atCNE presence (purple), and absence of older (Vertebrate) atCNE.

We used the zCNE and fCNE datasets as reference CNE queries to scan the other teleost genomes via BLAST, while we also included the spotted gar genome as an outgroup to identify CNE gains and losses following the 3R WGD (supplementary tables S2 to S4 and S6, Supplementary Material online). This approach allowed us to infer the ancestral teleost CNE set, by using presence/absence information of zCNEs and fCNEs in different species of our phylogeny. Taking advantage of the phylogenetic positions of our selected focal species, we carried out a bidirectional comparison, reviewing the presence of fCNE in the Zebrafish–Mexican tetra clade (Clade 1) and the presence of zCNE in the Fugu–gilthead seabream clade (Clade 2), as described in more detail in the related Materials and Methods section and summarized in Fig. 1a. This search identified 7,358 fCNEs present in at least one species of Clade 1 and 22,596 zCNEs present in at least one species of Clade 2. Combining these sets produced a final set of 25,930 nonoverlapping unique elements that consists the ensemble of CNEs inherited by at least two or more species in our phylogeny from the teleost common ancestor (atCNEs).

### Extensive CNE Gain and Loss During Early Teleost Evolution

We next used the reconstructed atCNE set to review previously reported CNE loss in teleosts and to assess putative gains during early teleost evolution. We identified 6,585 atCNE that are also found in other vertebrates (Vertebrate atCNE), 6,934 atCNE that are shared only with spotted gar and no other vertebrates (Neopterygian atCNE), and 12,411 atCNE with no hit in vertebrates or spotted gar and are teleost specific (3R atCNE) (Fig. 1a, supplementary table S7, Supplementary Material online). We also used publicly available sets of CNEs from the ANCORA database, combining nonoverlapping unique CNE shared by human-spotted gar and human-elephant ghost shark to obtain an ancestral Vertebrate CNE set (vCNEs). Searching for vCNEs in any of the teleost genomes in our phylogeny confirmed previously reported levels of loss of ancestral vCNEs in teleosts (69.5%) (supplementary tables S5 and S6, Supplementary Material online). A characteristic of vertebrate CNEs is their nonrandom distribution in the genome, with many clustering around developmental TF genes. To assess if gained atCNEs follow a similar distribution pattern, we looked into the distribution of the three different categories of atCNEs in the zebrafish genome. After dividing the zebrafish genome in 500 kb nonoverlapping windows (hereinafter referred to as 500 kb zones), we counted total CNE content for each category in each zone (supplementary table S8, Supplementary Material online). Of the total 2,664 500 kb zones, 1,982 zones have atCNE presence, with 985 of these zones containing vertebrate atCNEs, 1,179 zones containing Neopterygian atCNEs and 1,690 zones containing 3R atCNEs, while 682 zones have absence of atCNEs of any category (Fig. 1b).

### Evaluating atCNEs as Phylogenetic Markers

**Fig. 2.**
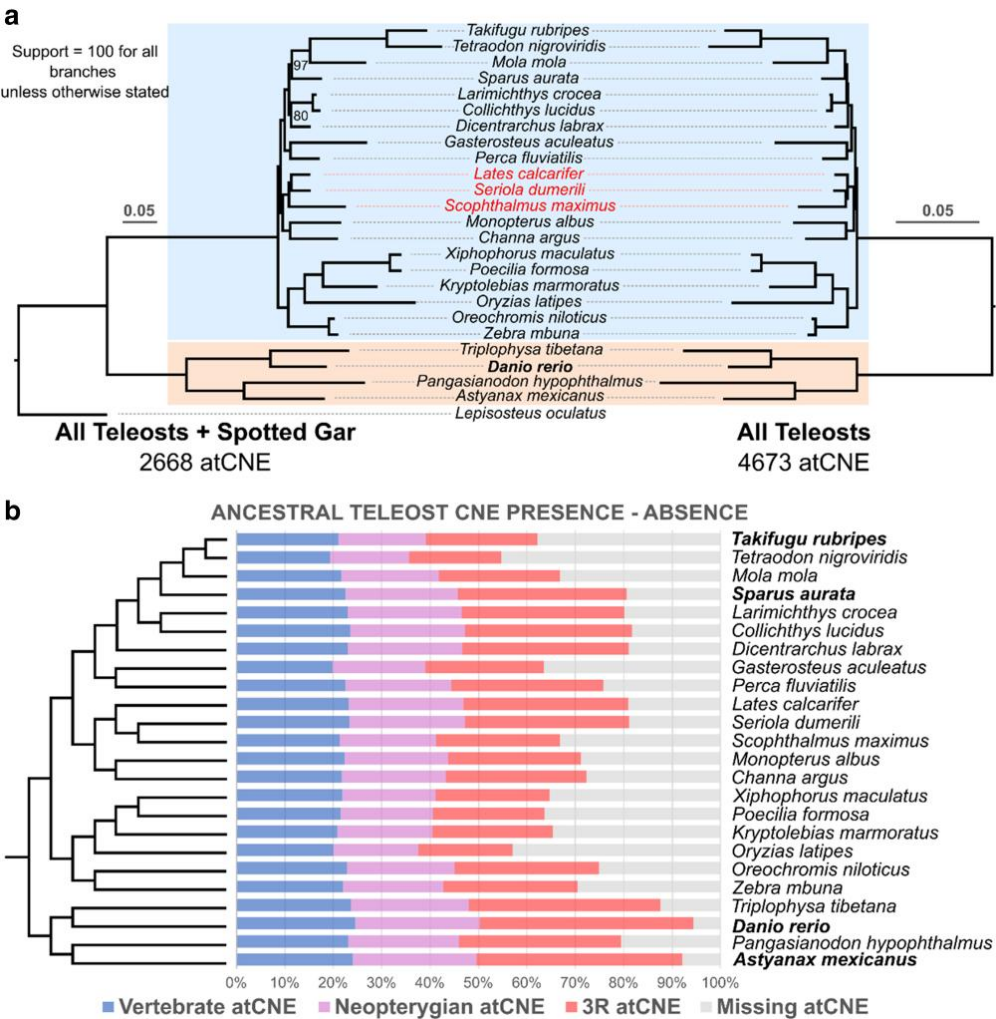
Ancestral CNE losses across the teleost phylogeny. a) Phylogenetic reconstruction of the relationships of the 24 teleost species included in the study, using single copy universal atCNEs, including (left tree—2,668 atCNEs) or excluding (right tree—4,673 atCNEs) spotted gar. The two basally diverged clades used to infer ancestral teleost CNEs are highlighted: clade 1 (from *A. mexicanus* to *T. tibetana*) and clade 2 (from *Z. mbuna T. rubripes*). Branches with species highlighted in red show topological discrepancies in the spotted gar shared atCNE phylogeny (left). b) Presence/absence of ancestral atCNE categories of different origin (Vertebrate in blue, Neopterygian in purple, 3R in red) across the teleost phylogeny as a percentage of total identified atCNEs. The cladogram is constructed based on phylogenetic reconstructions from (a).

Taking advantage of our atCNE set, we assessed the potential of atCNEs as phylogenetic markers. We constructed two CNE-based phylogenies, one with an extended dataset of 4,673 pan-teleost atCNEs present in all teleost species in the study but excluding spotted gar and one from a subset of the pan-teleost dataset with 2,668 elements also present in spotted gar. In parallel, we also constructed a protein-based phylogeny using universal single copy ortholog genes as a control for assessing resulting topologies from the CNE-based reconstructions. Use of the pan-teleost dataset of 4,673 atCNEs lead to the recovery of the expected topology, as supported by the gene-based tree (supplementary fig. S3, Supplementary Material online), with maximal bootstrap support for all clades (Fig. 2a). In contrast, the dataset including spotted gar resulted in topological discrepancies in the relationships of *Lates calcarifer*, *Seriola dumerili*, and *Scophthalmus maximus* and lower support in some of the clades (highlighted in red in Fig. 2a).

### Variable Ancestral CNE Loss across the Teleost Phylogeny

To characterize patterns of atCNE gain and loss in different teleosts, we mapped rates of loss for each atCNE category (supplementary table S9, Supplementary Material online) as a percentage of all identified atCNEs, as presented in Fig. 2b. A range of CNE loss is seen across the phylogeny, with different species having variable atCNE retention levels (Fig. 2b), while different rates of loss were found for different atCNE categories. Vertebrate atCNEs have the lowest rates of loss ranging from 0.84% to 6.09%, with an average loss of 3.18% (standard deviation = 1.33%). In turn, Neopterygian atCNEs show nearly 2-fold loss in comparison, ranging from 1.08% to 10.53%, with an average loss of 5.31% (standard deviation = 2.56%). Finally, 3R atCNEs account for two-thirds (average 68.1%) of total atCNE loss, with their loss ranging from 3.67% to 28.75% of all atCNEs, with an average of 17.82% (standard deviation = 6.78%).

### High Sequence and Synteny Conservation across Teleost CNE

**Fig. 3.**
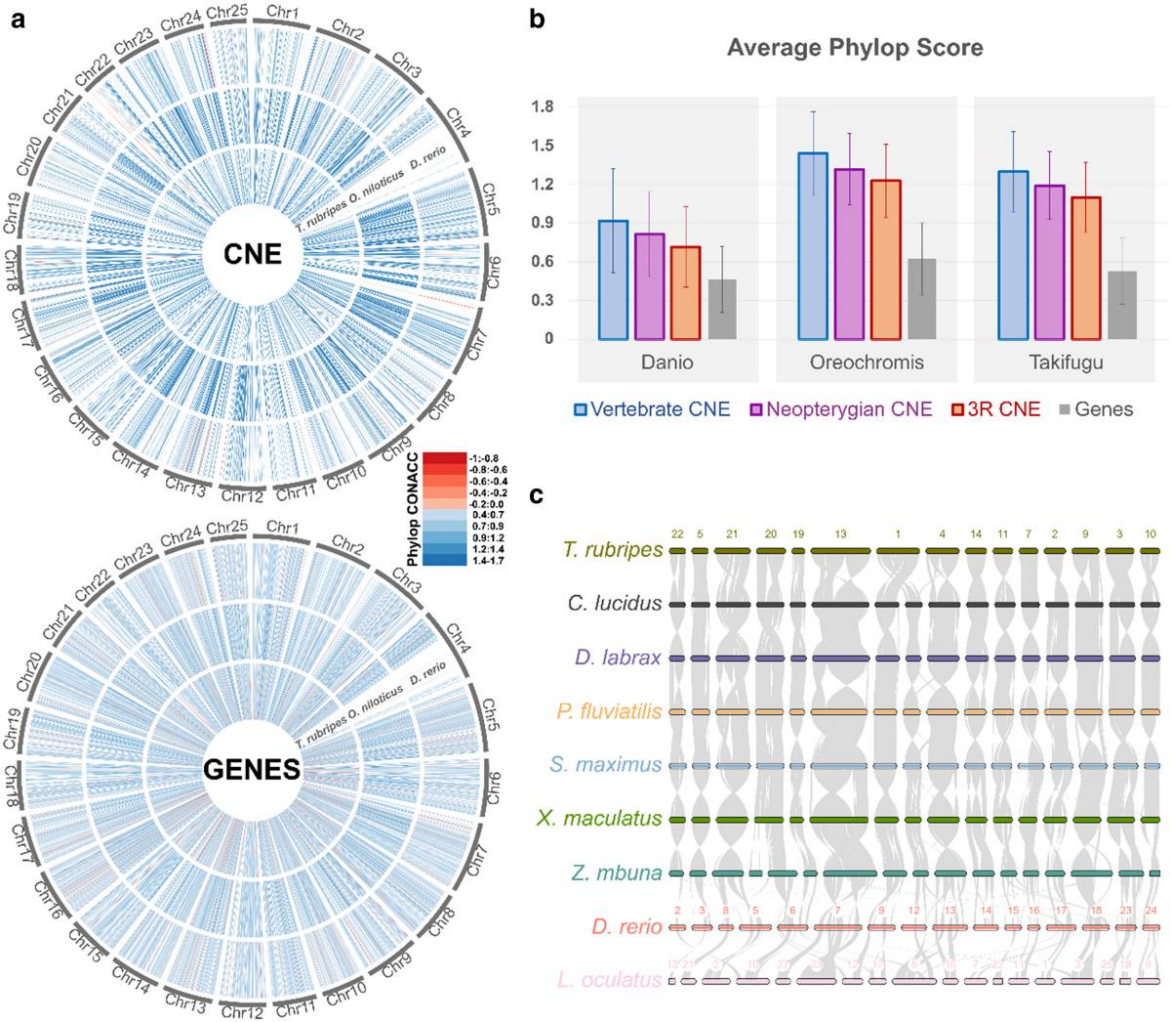
CNE sequence and synteny conservation. a) PhyloP (CONACC) conservation scores for atCNEs and Genes in *D. rerio*, *O. niloticus*, and *T. rubripes* mapped on *D. rerio* chromosomes. b) Average phyloP (CONACC) conservation scores and standard deviation for atCNEs in different categories and genes in *D. rerio*, *O. niloticus*, and *T. rubripes*. c) Synteny of atCNEs across eight teleost genomes and *L. oculatus*.

Vertebrate CNEs are characterized by high levels of sequence conservation, being on average more conserved than exonic areas (Polychronopoulos et al. 2017). To assess to what extent different categories of teleost CNEs follow this pattern of slow evolution, we carried out multiple whole-genome alignment of the genomes included in our phylogeny and calculated phyloP conservation scores for atCNEs in Zebrafish, Fugu, and Nile Tilapia (supplementary tables S10 to S13, Supplementary Material online). We selected Zebrafish and Fugu as the focal species used for zCNE and fCNE identification, respectively. As a third species we wanted to include a member of the major branch of Clade 2 that spans from *Xiphophorus maculatus* to *Zebra mbuna* in Fig. 2a, instead of the branch that includes the Fugu, spanning from Fugu to *C. argus* in Fig. 2a. We chose the tilapia as the species with the lowest levels of CNE loss from that branch. We also assessed CNE synteny in chromosome level genomes included in our phylogeny, hypothesizing that the clustered distributional pattern of the majority of atCNEs around specific genomic regions suggested high synteny conservation. Teleost CNEs have significantly higher average phyloP scores compared to exonic sequences. What is more, vertebrate atCNEs have higher average phyloP scores than Neopterygian atCNEs, which in turn have higher phyloP scores than 3R atCNEs (Fig. 3b). CNE synteny is also largely conserved across teleosts, with larger syntenic areas shared among Clade 2 species and notable rearrangements compared to zebrafish and spotted gar (Fig. 3c; supplementary fig. S4, Supplementary Material online).

### Gene Association and Paralog Analysis Highlight Teleost CNE Evolution

**Fig. 4.**
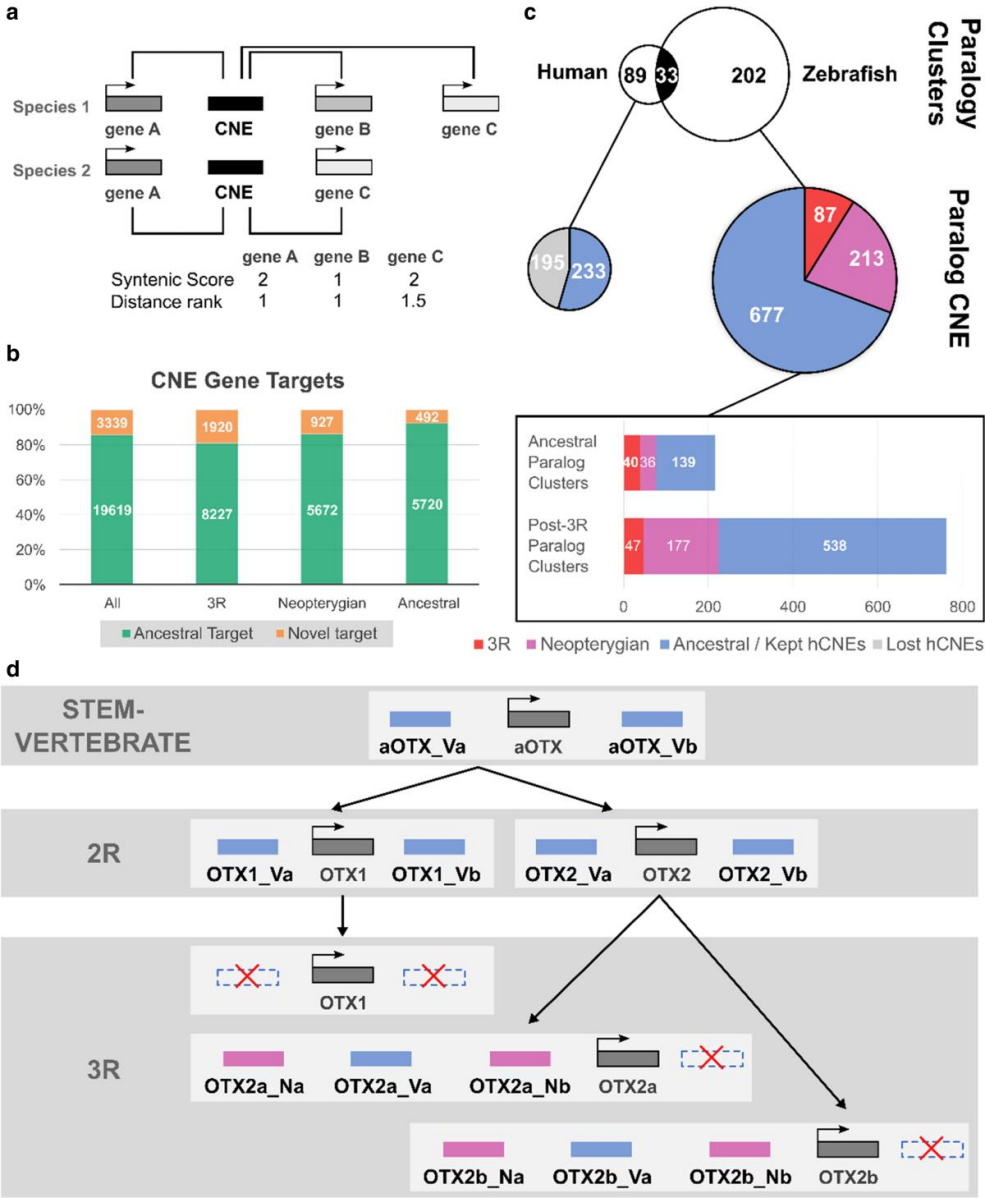
CNE–gene target association and paralog analysis. a) CNE were linked to putative target genes through orthology-guided synteny-based association, using the maximal syntenic score (number of species a CNE–gene pair is found within 1 Mb) and minimal proximity rank (position of a gene to a CNE when all genes are ordered in increasing distance) for each CNE-target pair. b) Percentage (and counts within boxes) of ancestral (atCNE also proximal to orthologous locus in human) or novel gene targets for atCNEs in different categories. c) Paralogy cluster and paralog CNE counts for different categories in human/other vertebrates (left) or zebrafish/teleosts (right). Common paralogy clusters between the two groups are shared in black and common/ancestral atCNEs within paralogy clsuters are shown in blue. d) CNE gains, duplications and losses in the *OTX1*/*OTX2* paralogous loci in vertebrates and teleosts. The ancestral *OTX* locus in stem-vertebrate *aOTX* had two ancestral vertebrate CNEs (aOTX_Va & aOTX_Vb). Paralogous copies of these elements are found in human *OTX1* (OTX1_Va & OTX1_Vb) and *OTX2* (OTX2_Va & OTX2_Vb). In zebrafish, OTX1_Va, OTX1_Vb, and OTX2_Vb were lost and the 3R WGD produced two paralogous copies of OTX2_Va (OTX2a_Va & OTX2b_Va). Two Neopterygian gained CNEs in the *OTX2* locus (OTX2_Na & OTX2_Nb) also gave rise to two pairs of paralogous elements in the paralogous loci of *OTX2a* (OTX2a_Na & OTX2a_Nb) and *OTX2b* (OTX2b_Na & OTX2b_Nb).

Since CNEs have been shown to act as conserved developmental enhancers, we undertook a synteny-based association approach to identify putative gene targets (Fig. 4a). Briefly, for each gene within 1 Mb upstream or downstream of a zCNE or fCNE we attributed a synteny score (the number of species in which the CNE–gene pair is found) and a proximity rank (average position of each gene from a CNE in increasing order of distance). One or more targets (in case of tied candidates) with the highest syntenic score and the lowest distance rank were linked to each CNE (Gene A in Fig. 4a for example), using a minimum synteny score threshold of five species (supplementary tables S14 to S18, Supplementary Material online).

Our gene association pipeline associated 20,168 zCNEs with 4,444 zebrafish genes and an additional 2,790 fCNEs not corresponding to zCNEs with 1,146 Fugu genes. Comparing these sets to vCNE associated genes in human revealed that 85.46% of all atCNEs with targets were associated with a locus that has a human ortholog located within 1 Mb of a vCNE. We termed loci with human orthologs associated with vCNE “ancestral targets” and loci without human orthologs associated with vCNEs “novel targets” (Fig. 4b). Looking at individual categories of atCNE with targets, 92.08% of vertebrate atCNEs, 85.95% of Neopterygian atCNEs, and 81.08% of 3R atCNEs were associated with ancestral targets (Fig. 4b). Gene ontology enrichment for ancestral atCNE targets showed high enrichment for genes involved in transcriptional regulation, the development of various organ systems, and neuronal specification (Table 1; supplementary tables S19 and S20, Supplementary Material online). Novel atCNE targets were mainly enriched for developmental processes already associated with ancestral target sets, but had significantly weaker enrichment support, while one process (regulated exocytosis) specifically enriched only in novel target genes (Table 1; supplementary tables S19 and S21, Supplementary Material online).

**Table 1:** Gene ontology enrichment for ancestral and novel CNE–gene target sets

term_id	term_name	Padj ancestral target	Padj novel target	Ancestral target examples	Novel target examples
GO:0032502	Developmental process	7.14E−48	1.60E−9	shox2,twist3,meis2a,smad1,tlx1	nectin1b,pbx4,foxo6b,elf1,gli1
GO:0007399	Nervous system development	9.48E−39	1.69E−2	pbx3b,wnt3,foxd3,irx2a,pax6b	neurod4,fgf24,notch3,irx7,meis3
GO:0030154	Cell differentiation	1.22E−28	3.64E−7	gata6,smad6b,nr2f2,prdm8b,fgf4	smad4a,foxd2,myog,wnt6b,nkx3.3
GO:0022008	Neurogenesis	4.75E−26	5.56E−3	LHX3,neurod2,prox1a,fgf19,irx1a	wnt11,ntrk1,sema4c,sox10,anxa1a
GO:0007166	Cell surface receptor signaling pathway	3.24E−6	1.32E−2	lef1,wnt8b,fgf20b,her6,DTX1	pappa2,sema3fa,stat6,nod2,fgf22
GO:0007267	Cell–cell signaling	1.05E−5	4.89E−3	gbx1,axin2,panx3,nkx6.1,cacna1ba	lbx2,wnt8a,tet3,GRM7,cacng8b
GO:0007169	Transmembrane receptor protein tyrosine kinase signaling pathway	1.82E−4	2.41E−2	zeb2a,ERBB4,fgfr2,ntrk2b,nrg1	efna3a,ltk,fgfrl1a,ntrk1,fgf22
GO:0010468	Regulation of gene expression	1.29E−23	…	dmbx1a,foxf2a,tbx2b,barx2,gsx1	…
GO:0060429	Epithelium development	1.07E−12	…	tbx21,glis2b,runx2b,tfap2c,gata5	…
GO:0007167	Enzyme-linked receptor protein signaling pathway	7.12E−9	…	ntrk2b,pappaa,fgf10a,tgfb3,dusp6	…
GO:0070848	Response to growth factor	6.95E−8	…	fgfr2,tgfbr3,smad7,vegfc,fgf1b	…
GO:0003002	Regionalization	1.64E−7	…	bmb2b,dmrt2a,tcf7,sox17,vent	…
GO:0007423	Sensory organ development	3.34E−7	…	pbx1a,irx2a,six3a,meis1b,hmx1	…
GO:0035295	Tube development	9.20E−7	…	fgf10a,twist1a,sox7,onecut1,meis1b	…
GO:0030900	Forebrain development	9.67E−6	…	prox1a,eya1,pax6b,gli2a,nkx2.1	…
GO:0072359	Circulatory system development	9.79E−6	…	smarca5,unc5b,mef2aa,twist1a,foxc1a	…
GO:0007507	Heart development	1.10E-4	…	enc3,fxr1,pou4f1,tbx19,dkk1b	…
GO:0048839	Inner ear development	1.16E−4	…	pax2a,hmx2,eya2,bmp2b,esrrga	…
GO:0048732	Gland development	2.00E−4	…	rpl3,dhpsnr5a2,xbp1,met,prickle1a	…
GO:0001501	Skeletal system development	8.87E−4	…	col1a2,faf1,bcl9,egf,ostn	…
GO:0045055	Regulated exocytosis	…	4.18E−2	…	otofa,SYT2,cacna1g,rims3,unc13ba

To identify WGD-derived CNE copies, we combined self-BLAST searches of zebrafish and Fugu atCNEs with paralogy information from ENSEMBL, while applying the same methodology to human vCNEs in parallel, to compare vCNE and atCNE paralogous loci and probe ancestral CNE fate after the 2R and 3R WGD (supplementary tables S22 to S25, Supplementary Material online). Our analysis identified 89 vCNE paralogy clusters in human and 202 atCNE paralogy clusters in zebrafish, with 33 common paralogy clusters shared by both groups (Fig. 4c). Of the total 977 paralogous atCNEs, 69.3% are vertebrate atCNEs, 21.8% are Neopterygian atCNEs, and 8.9% are 3R atCNEs, while 195 of 428 paralogous vCNEs were lost in teleosts (Fig. 4c). A total of 215 atCNEs (22%) are located in ancestral (2R derived) paralog clusters, with the remaining 762 atCNEs (78%) found in 3R derived paralog clusters (Fig. 4c). As an illustrative example of paralogous CNE evolution, gains and losses of paralogous CNEs in the *OTX1*/*OTX2* loci are presented in Fig. 4d. Based on the structure of the locus in teleosts and other vertebrates, the ancestral *OTX* locus in stem-vertebrates (*aOTX*) contained two CNEs (aOTX_Va and aOTX_Vb), with each giving rise to a paralogous element in the paralogous loci of *OTX1* (OTX1_Va & OTX1_Vb) and *OTX2* (OTX2_Va & OTX2_Vb) after the 2R WGD. In teleosts, OTX1_Va, OTX1_Vb, and OTX2_Vb were lost, while OTX2_Va is found in two paralogous copies (OTX2a_Va–OTX2b_Va) in the paralogous *OTX2a* and *OTX2b* loci. In parallel, two CNEs gained in the *OTX2* locus in the Neopterygian ancestor (OTX2_Na and OTX2_Nb) gave rise to two pairs of paralogous elements in the *OTX2a* (OTX2a_Na and OTX2a_Nb) and *OTX2b* (OTX2b_Na and OTX2b_Nb) loci after the 3R WGD as well.

### Gained and Ancestral Teleost CNE Share Similar Motif Vocabularies

**Fig. 5.**
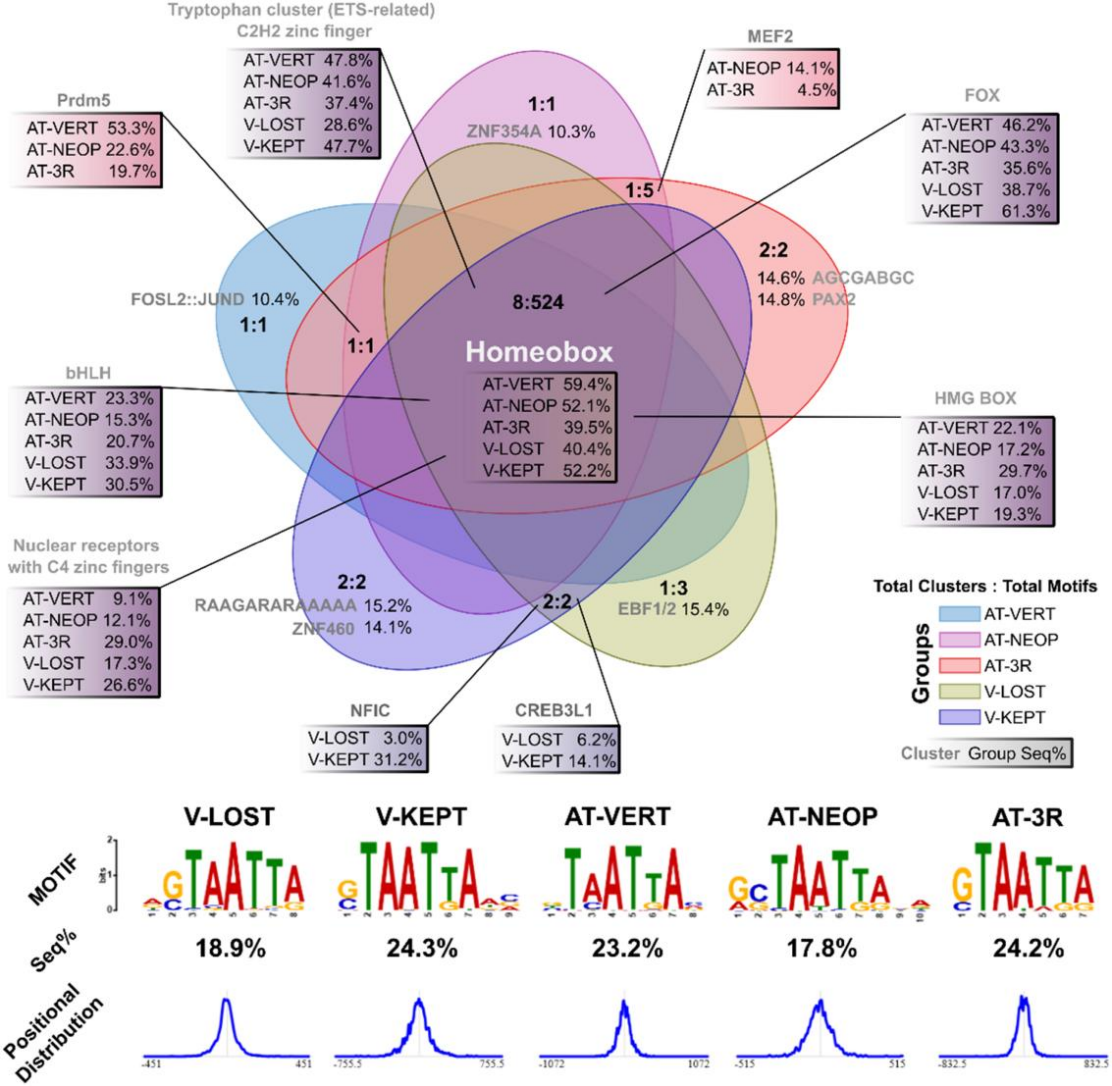
Motif discovery and TFBS enrichment. Top) Overlap of enriched TFBS categories among combinations of atCNEs (AT-VERT: Vertebate—blue, AT-NEOP Neopterygian—purple, AT-GAIN: 3R—red) and avCNEs (V-KEPT: Kept in Teleosts—dark purple, V-LOST: Lost in Teleosts—yellow). Enrichment overlaps are displayed in the format “Total Categories: Total Enriched Motifs” in each overlap area. The percentage of sequences of each CNE group with motifs in representative categories (c1, c2,…) are shown. Bottom) Percentage of sequences in each CNE group that contain the most common shared de novo predicted motif (consensus TAATTA) and its positional distribution.

To assess if gained atCNEs may be divergent ancestral elements, we carried out motif and transcription factor binding site (TFBS) enrichment on different sets of atCNEs (Vertebrate, Neopterygian, 3R) and vCNEs (Lost and Kept in Teleosts) (supplementary tables S26 to S32, Supplementary Material online). In parallel, as a control, we also carried out the same enrichment analysis on 10,932 random non-CNE zebrafish enhancers with similar size distribution to our CNE sets (supplementary table S33, Supplementary Material online). All CNE categories, but not non-CNE enhancers, were found to share a common TFBS vocabulary, dominated by the homeobox (40% to 60% of CNE), FOX (35% to 61%), EST-related/C2H2 zinc finger (28% to 47%), and bHLH (15% to 33%) classes (Fig. 5). De novo motif discovery also found an enrichment for the “TAATTA” homeodomain-binding motif, centrally positioned in 18% to 24% of all CNEs (Fig. 5). No shared enriched motifs were found between any atCNE and vCNE combinations, but 19.7% to 53.3% of atCNEs contained Prdm5 sites, which were not enriched in vCNEs (Fig. 5). Additionally, 14.8% of 3R atCNEs contained PAX2 sites, which were not specifically enriched in any other CNE category.

## Discussion

This work presents the first attempt to catalogue the previously undocumented variety of Neopterygian and teleost CNEs by using a teleost-centric identification protocol.

### Properties of Teleost CNE

Core aspects of the identity and evolution of teleost CNE are highlighted here. Importantly, our work shows that the loss of ancestral CNEs in early teleosts was accompanied by extensive gain of new elements. While a large number of new CNEs (35.8%) were gained in the Neopterygian ancestor, the majority of new elements (64.2%) were acquired after the split with spotted gar, following the 3R WGD. The study of the genomic distribution of ancestral and gained elements in zebrafish also provides further insight into how these elements were acquired. The majority of genomic zones containing vertebrate atCNEs (89.2%) also contain gained CNEs, with 80.4% of Neopterygian atCNEs and 84.7% of 3R atCNEs found in ancestral vertebrate zones. Respectively, novel zones without vertebrate atCNEs have much lower total CNE content, with novel Neopterygian zones (40.2% of all zones with Neopterygian atCNEs) containing only 19.6% of all Neopterygian atCNEs and novel 3R zones (30.9% of all zones with 3R atCNEs) containing only 15.3% of all 3R atCNEs. In addition, a quarter of the zebrafish genome (25.6%, i.e. 682/2,664 zones) is devoid of CNEs, supporting that all categories of teleost CNEs (whether ancestral or gained) exhibit nonrandom clustered distribution, which is distinct from the more homogeneous distribution of genic regions (Fig. 3a).

Overall, not just vertebrate atCNEs, but all teleost atCNEs exhibit properties that are comparable to those of CNEs from other vertebrates shown in previous studies: (1) they are distributed over the similar overlapping genomic regions, regardless of their time of origin, (2) they show high sequence and synteny conservation over large evolutionary distances (Kikuta et al. 2007; Polychronopoulos et al. 2017), (3) they are syntenically associated with loci involved in development related processes and TF genes and (4) they are enriched for development-related TFBS, with many harboring homeodomain binding motifs centrally in their sequence. The latter characteristic is in line with previous reports of AT enrichment in the conservation core of vertebrate CNEs, suggested to emerge from an overrepresentation of homeodomain binding motifs (Walter et al. 2005; Chiang et al. 2008).

### The Evolutionary Dynamics that Shaped the Teleost CNE Repertoire

The massive loss of ancestral CNEs in teleosts has been considered a mystery since early studies of vertebrate CNEs, mainly because the expected impact of the 3R WGD would be an addition of new paralogous CNEs to existing elements incrementally. This would be in line with models of CNE evolution via gradual turnover over time, with new CNEs replacing older elements at different rates. Under such a model, relatively steady rates of CNE loss and gain could be predicted at comparable evolutionary distances in different branches. Consequently, finding that novel CNEs gained in stem-teleosts have highly similar characteristics as ancestral vertebrate elements is pivotal for advancing our understanding of CNE evolution. There-establishment of the CNE repertoire following the 3R WGD is comparable to the emergence of ancestral CNEs in the jawed vertebrate common ancestor. With only few hundreds of CNEs shared with lamprey and only tens of elements recognizable in amphioxus, the vast majority of thousands of jawed vertebrate CNEs emerged only after the second WGD in jawed vertebrates (McEwen et al. 2009). CNE associated targets, as well as experimentally confirmed regulated targets, include loci involved in the development of the central and peripheral nervous systems, as well as heart and muscle during the specification of these tissues at the phylotypic stage (Woolfe et al. 2005). However, most of the innovations related with these cell populations and their associated gene regulatory networks predate the split of jawed vertebrates and are shared with cyclostomes. Similarly, we observed that the majority of teleost gained CNEs are also associated with these ancestral targets involved in developmental regulation at the phylotypic stage. This is in contrast with younger clade-specific CNE gains in mammals, which have been associated with novel target loci, many of which are involved in protein binding (Takahashi and Saitou 2012; Babarinde and Saitou 2013). Similarly, previous work showed that older vertebrate CNEs are implicated in transcriptional and developmental regulation, while younger mammalian elements are associated with genes involved in post-transcription modification (Lowe et al. 2011). Finally, gained (either Neopterygian or 3R) teleost CNE also share a highly similar motif and binding site composition as vertebrate CNE, supporting they are also regulated by similar upstream networks. Consequently, we hypothesize that the majority of gained CNEs in teleosts represent a reconfiguration of the ancestral vertebrate CNE repertoire linked to the regulation of the same key ancestral targets, instead of components of a new network regulating teleost specific processes.

### CNE Gain by Sequence Divergence and De Novo Emergence

**Fig. 6.**
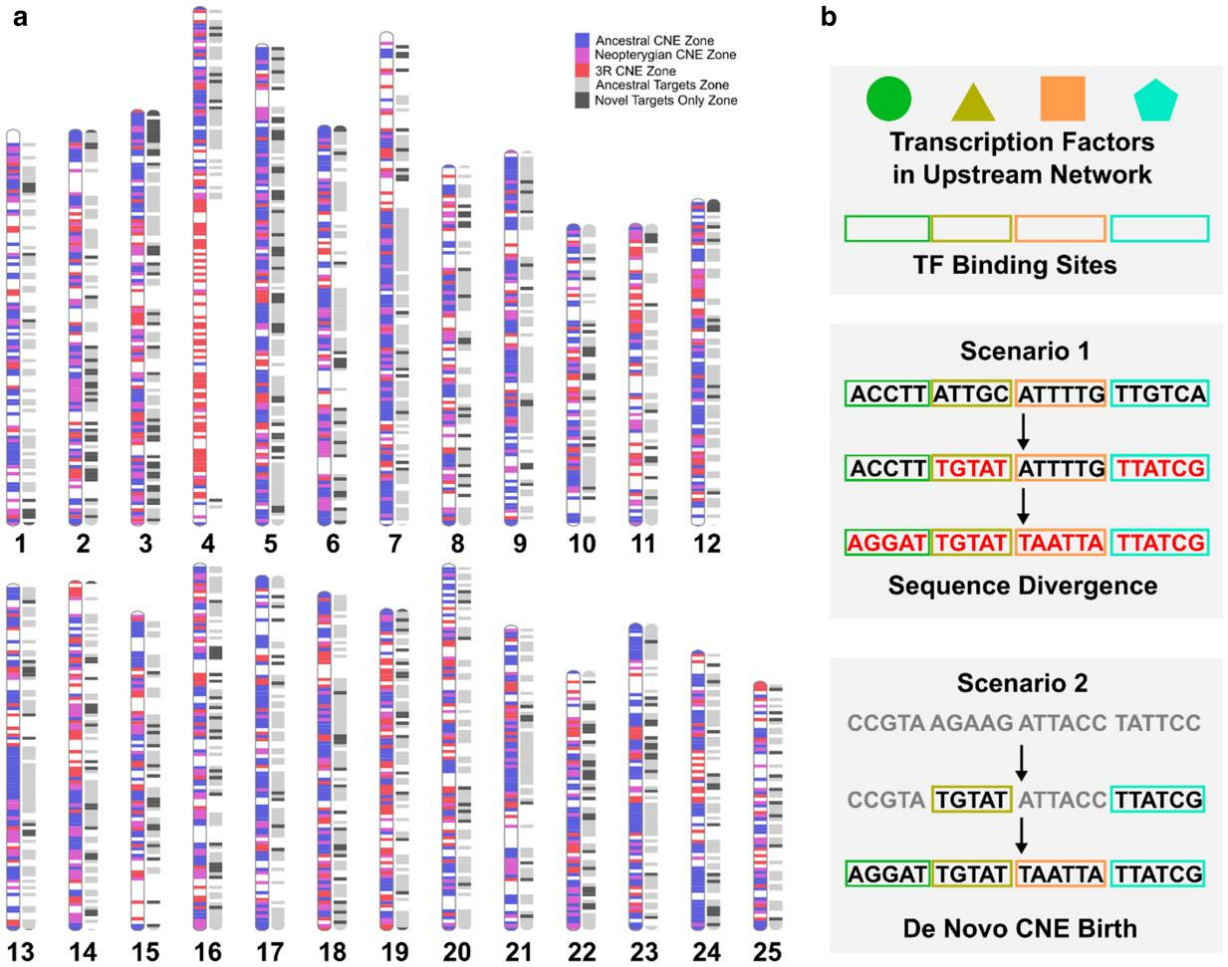
Teleost CNE-target localization and models of CNE gain. a) Localization of atCNEs and associated target genes in zebrafish chromosomes. CNE zones are plotted in blue if they contain vertebrate atCNEs, in purple if they contain Neopterygian atCNEs and do not contain vertebrate atCNEs, in red if they contain 3R atCNEs and do not contain any older (vertebrate or Neopterygian) atCNEs. Gene zones are plotted in light gray if they contain ancestral targets or in dark gray if they contain only novel targets and do not contain ancestral targets. b) Two hypothesized scenarios of atCNE gain: scenario 1: Extreme sequence divergence of ancestral sequence renders CNE unrecognizable, while TFBS are preserved and scenario 2: CNE gain through de novo emergence.

While atCNEs of different origin categories have largely comparable characteristics, there are differentiating features that are detected when comparing gained (Neopterygian or 3R) and ancestral (vertebrate) teleost CNE. When considered in decreasing age of conserved sequence fixation (Vertebrate > Neopterygian > 3R), older atCNEs show higher average conservation, have lower loss rates across the phylogeny and are associated with more ancestral target genes compared to newer elements. These differences may originate from a small number of Neopterygian and 3R atCNEs that are located within novel genomic regions without vertebrate atCNEs and which are associated with novel teleost-specific targets (Fig. 6a). Based on this deviating profile compared to other atCNEs, it can be suggested that these novel-target associated elements are cases of de novo CNE emergence, as part of teleost-specific regulatory networks. We, therefore, hypothesize that the ensemble of the CNEs gained in the stem-teleost lineage derived from two complementary scenarios of evolutionary origin (Fig. 6b). The homogeneous profile of the majority of atCNEs is compatible with a scenario of CNE gain through extreme sequence divergence, in which ancestral vertebrate elements deviate in primary sequence, while retaining ancestral TFBS content and remaining associated with ancestral targets. This must have then been coupled with de novo gains, as supported by both the emergence of novel-target teleost specific elements and the evolution of Neopterygian CNEs around ancestral targets prior to the extensive CNE loss in teleosts. The analysis of paralogous CNE evolution further illustrates this, with more than one in five paralogous atCNEs having Neopterygian origin (21.8%), as in the case of the *OTX1*/*OTX2* loci where gain of Neopterygian paralogous atCNEs was coupled with loss of ancestral vertebrate paralogous CNEs.

## Materials and Methods

To identify teleost CNEs we used the identification pipeline described below and presented in supplementary fig. S1, Supplementary Material online. Briefly, we first selected 24 teleost genomes and the spotted gar genome from the Ensembl database (Cunningham et al. 2022) (supplementary table S1, Supplementary Material online), aiming for high contiguity (N50 > 8 Mb), high completeness (BUSCO score of >95%) and broad sampling across the teleost phylogeny. We then used pairwise whole-genome alignments from two selected pairs of focal reference species for de novo CNE identification and used these starting CNE sets to identify ancestral teleost elements, as well as CNE gains and losses across the phylogeny.

### Pairwise Whole-genome Alignment

We performed pairwise reciprocal whole-genome alignments with LASTZ v1.04.03 (Harris 2007) for two focal reference pairs of genomes downloaded from the Ensembl database (Howe et al. 2021), *Danio rerio* (Zebrafish—DanRer11) vs *Astyanax mexicanus* (Mexican tetra—Astyanax_mexicanus-2.0) and *Takifugu rubripes* (Fugu—fTakRub1.2) vs *Sparus aurata* (Gilthead seabream—fSpaAur1.2). These pairs were selected as representatives of the two most basally splitting clades of teleost phylogeny (206 to 252 MYA [Kumar et al. 2017]), to allow for inference of ancestral CNE sets through comparisons across the phylogeny. All genomes were downloaded hard masked, i.e. interspersed repeats and low complexity regions were detected with the RepeatMasker tool (Tarailo-Graovac and Chen 2009) and were replaced with N. To conduct the alignments, we split input genomes in 10 Mb windows with 100 kb overlaps for Zebrafish and Fugu and non-overlapping 10 Mb windows for Mexican tetra and gilthead seabream. Two rounds of pairwise whole-genome alignments were run for each pair as discussed by Hiller et al. (2013). In the first round, we used the following more sensitive LASTZ parameterization recommended for distantly related species (>100 MYA): *M* = 50, *E* = 30, *H* = 2,000, *K* = 2,200 *L* = 6,000, *O* = 400, *T* = 1, *Y* = 3,400, *Q* = HoxD55.q (Tan et al. 2019). Subsequently, a second round of alignment was performed after masking aligned regions from the first alignment round, to facilitate the identification of conserved elements not found in the initial alignment. During the second round, the following less sensitive LASTZ parameters were used: *K* = 1,500, *L* = 2,300, *M* = 0, and *W* = 5 (Hiller et al. 2013).

### Chaining—Netting

Aligned regions found by the two rounds of pairwise whole-genome alignment were chained using the CNEr v1.8.3 Bioconductor wrapper functions (Tan et al. 2019) inside an R environment (using utilities found in UCSC Browser [Kent et al. 2002]). During chaining, matching sufficiently close initial alignments are joined into bigger alignment chains, as described by Tan et al. (2019) (Kent et al. 2002), with the longest fragments further connected to form netted alignments in net Axt format (supplementary fig. S1, Supplementary Material online).

### CNE Identification in Reference Focal Species

Alignment chain nets from the two focal reference pairs (Zebrafish vs Mexican tetra and Fugu vs Gilthead seabream) were used for de novo CNE identification using Zebrafish and Fugu as reference genomes. Exonic regions were filtered out of alignments, using gene annotation information from the Ensembl database (*Takifugu_rubripes.fTakRub1.2.104*, *GCF_900880675.1_fSpaAur1.1_genomic*, *Astyanax_mexicanus-2.0.104*, *Danio_rerio.GRCz11.104*). The CNEr v1.28.0 Bioconductor package (Tan et al. 2019) was used for both filtering and CNE detection. Conserved regions were selected using the following parameters: (1) a maximum number of four hits per element (cutoffs = 4), i.e. how many times we expect to see an element, (2) a minimum identity of 70% for aligned regions (Identities = 70pc), i.e. the minimum percentage of matches in a single alignment, and (3) a sliding window size of 100 bp for detection (windows = 100).

### CNE Search in Other Teleost Species

Using the de novo identified CNE datasets from Zebrafish (*zCNEs*) and Fugu (*fCNEs*), we searched 20 other teleost fish genomes premasked for repeats via BLAST v2.10.0+ (Altschul et al. 1990) for zCNE and fCNE presence/absence (e-value <= 1e^−6^, word size = 6, max target seqs = 1, max hsps = 1). This comparative dataset was used as the basis for studying CNE conservation, gain, and loss, as well as CNE-based phylogenomic analyses.

### CNE-based Phylogenomic Analysis

For CNE-based phylogenomic reconstructions, we used single copy CNEs shared by all species in the phylogeny, including or excluding spotted gar (*Lepisosteus oculatus*) as an outgroup species belonging to the Holostei infraclass of ray-finned bony fish, which diverged before the 3R WGD (Braasch et al. 2016). To extract CNE sequences for multiple alignments from different teleost or spotted gar, we used BLAST derived coordinates extended by 50 bp in either side to maximize alignable sequence capture (supplementary fig. S2, Supplementary Material online). Multiple sequence alignments were carried out using MAFFT v7.407 (Katoh et al. 2002) and CNE alignments were collated with custom bash scripting to produce a single supermatrix of all CNE from across all species in each phylogeny. TrimAl v1.4.rev15 (Capella-Gutiérrez et al. 2009) was used for alignment trimming with a gap threshold of 50% (>0.5). RAxML-NG v.1.0.2 (Stamatakis et al. 2003) was used for CNE-based tree construction using 20 starting trees (10 parsimony + 10 random) and 100 bootstraps, with TVM + I + G4 selected as the best-fit model with ModelTest-NG (Darriba et al. 2020). For visualization purposes, FigTree v1.4.4 (Rambaut 2009, 2016) was used. Gene-based reconstruction was carried out with the same pipeline, using single copy orthologous proteins for all species included predicted by OrthoFinder2 v2.5.4 (Emms and Kelly 2015) from the proteomes of these species. Protein sequences were aligned with MAFFT v7.407, collated to a supermatrix with subsequent trimming using TrimAL v1.4.rev15 (>0.5). Tree construction was performed with RAxML-NG v.1.0.2 using 20 starting trees (10 parsimony + 10 random) and 100 bootstraps, with JTT + I + G4 + F selected as the best-fit model with ModelTest-NG.

### Identifying the Vertebrate CNE Set

For inference of ancestral vertebrate CNE losses in teleosts, CNE datasets for *Homo sapiens* (Human), *Callorhinchus milii* (Elephant shark), and *L. oculatus* (Spotted gar) (using Human as reference) were downloaded from the ANCORA database and were re-filtered for coding regions (*H. sapiens* gene predictions from RefSeq, Genbank, CCDS, UniProt. and the UCSC KnownGene track), selecting sequences with >70% identity over a window of 100 bases. These ancestral vertebrate CNE were then searched in the 24 teleost genomes in the study using BLAST (e-value = 1e^−6^, word size = 6, max target seqs = 1, max hsps = 1).

### Ancestral Teleost CNEs Identification and Categorization

We based our ancestral CNE identification protocol on comparisons between the two earliest splitting teleost clades in our phylogeny hereby defined as: clade 1 (highlighted in orange in Fig. 2a) spanning from *Siluriformes* represented by *Pangasianodon hypophthalmus* to *Cypriniformes* represented by *D. rerio* and clade 2 (highlighted in blue in Fig. 2a) spanning from *Cyprinodontiformes* represented by *Kryptolebias marmoratus* to *Tetraodontiformes* represented by *Mola mola.* To infer the ancestral teleost CNE set (from now on abbreviated as atCNEs), we selected zCNEs with presence in at least one “clade 2” species and fCNEs (not already overlapping zCNEs) with presence in at least one “clade 1” species, as these elements would have been inherited from the common ancestor of both clades (the teleost common ancestor).

We then searched for atCNEs presence in spotted gar or other vertebrates (ghost shark, western clawed frog, chicken, green anole, and human) via BLAST (e-value = 1e^−6^, word size = 6, max target seqs = 1, max hsps = 1) and allocated atCNEs to the following categories based on their inferred time of origin: (1) Elements identified in any vertebrate other than spotted gar were categorized as “vertebrate atCNEs”, inferred to be ancestral to ray-finned fish, (2) elements present in spotted gar and teleosts and absent from all other vertebrates were categorized as “Neopterygian atCNEs”, inferred to have been gained in the common Neopterygian ancestor with spotted gar, and (3) elements present only in teleosts were categorized as “3R atCNEs”, inferred to have been gained in the teleost common ancestor.

CNE distribution and localization over zebrafish chromosomes was plotted using Circos (Krzywinski et al. 2009) (Fig. 1b) and Rideogram (Hao et al. 2020) (Fig. 6).

### CNE Sequence and Synteny Conservation Analyses

To calculate CNE sequence conservation scores in three representative genomes from different parts of the phylogeny (*D. rerio*, *Oreochromis niloticus*, and *Takifugu rubripes*), we carried out multiple whole-genome alignments for all 25 species in the study with CACTUS (v 2.2.1) (Armstrong et al. 2020), applied phyloFit (Siepel and Haussler 2004) for phylogenetic model fitting (using the REV substitution model) for each chromosome for each species and then used phyloP (Pollard et al. 2010) to infer base-wise conservation scores (SPH method, CONACC mode). Next, we calculated average phyloP (CONACC) scores for zCNEs present in each species, as well as exonic areas, and plotted conservation scores over the 25 zebrafish chromosomes as a heatmap using Circos (Krzywinski et al. 2009). CNE synteny plotting for Fig. 3 was created using the JCVI MCscan pipeline (Tang et al. 2008).

### Gene Association and Ontology Enrichment

The association of CNEs with putative gene targets was carried out through an orthology-guided synteny-based approach. First, we extracted all genes that lie within 1 Mb upstream and downstream of each CNE in each teleost species. A synteny score was attributed to each CNE–gene pair for zCNEs (Zebrafish as reference), fCNEs (Fugu as reference), or vCNEs (human as reference) which corresponds to the number of species in which the CNE is proximal to an orthologous gene, based on orthology information for all genes across species obtained by OrthoFinder2 v2.5.4. We then calculated a proximity rank for each target gene, which corresponds to the average position of a gene relative to a CNE, when all proximal genes are ordered in increasing distance (Fig. 4a). Teleost CNEs were associated with candidate target genes with the highest synteny score (cutoff threshold of at least five species out of the 24 species scanned) and the lowest proximity rank (accepting all ties with the same syntenic score). For vCNE, we identified all target human genes within 1 Mb of each CNE that are syntenic in human and either spotted gar or elephant shark at minimum. Associated atCNE targets in zebrafish or Fugu were then compared to vCNE targets, using Orthogroup information from OrthoFinder2. Gene targets of atCNE with orthologous loci found within 1Mb of vCNE were annotated as “ancestral targets” and gene targets without orthologous loci close to vCNE were annotated as “novel targets”. Furthermore, we used a bootstrap resampling approach to obtain support that the observed levels of association with ancestral targets were non-random. zCNEs or fCNEs were randomly associated with subsets of genes sampled from all zebrafish or Fugu genes which were compared to vCNE associated loci and the total number of ancestral or novel target associated CNE was counted. Using 10,000 bootstrap replicates with different random subsets, we calculated the average count, standard deviation, and *z*-score for ancestral or novel target associated elements and obtained strong support (*z*-score <= 22) that associations were nonrandom. Gene ontology enrichment for GO biological process terms in different associated target gene sets was carried out through gProfiler (Raudvere et al. 2019).

### CNE Paralog Analysis

To identify CNE paralogs and clusters of paralogous CNE loci, we first carried out self-BLAST searches for zebrafish atCNEs and Fugu atCNEs to identify elements in each dataset with nonself-hits. Gene paralog information for each species was then acquired from the Ensembl database and used to annotate putatively paralogous atCNE loci (as inferred from BLAST matches) based on the paralogy of their associated target genes. Gene paralog information was also used to infer the origin of paralogous atCNE loci prior or after the 3R WGD, based on the clade of origin of paralogous genes. In parallel, the same pipeline was applied to human vCNEs and inferred paralogous vCNEs were then compared to paralogous atCNEs to identify common paralogy clusters between the two groups that have orthologous loci associated with paralog CNEs in both groups.

### Motif Prediction and Transcription Factor Binding Site Enrichment

De novo motif prediction and TFBS enrichment analysis for different CNE sets were carried out using XSTREME online from the MEME suite. Enriched TFBS were grouped to higher order categories based on similarity inferred from their consensus motifs and XSTREME motif clustering information. We also carried the same enrichment analysis on a subset of 10,932 random non-CNE zebrafish 24hpf embryonic enhancers downloaded from the EnhancerAtlas 2.0 database(Gao et al. 2016) with comparable size distribution to CNE sets as a reference for motif and TFBS composition in nonconserved developmental enhancers.

## Supplementary Material

evae061_Supplementary_Data

## Data Availability

Publicly available genomic data used for the analyses in this work are listed in supplementary data. Data generated as part of this study (including CNE genomic coordinates) are provided through the supplementary data document and supplementary tables S1 to S33, Supplementary Material online in supplementary files 1 to 5, with table description included in the supplementary data document. New code generated and used in this project will be made publicly available through https://github.com/vpapadog/teleost_CNEs.
